# Gene‐based mapping of trehalose biosynthetic pathway genes reveals association with source‐ and sink‐related yield traits in a spring wheat panel

**DOI:** 10.1002/fes3.292

**Published:** 2021-05-07

**Authors:** Danilo H. Lyra, Cara A. Griffiths, Amy Watson, Ryan Joynson, Gemma Molero, Alina‐Andrada Igna, Keywan Hassani‐Pak, Matthew P. Reynolds, Anthony Hall, Matthew J. Paul

**Affiliations:** ^1^ Computational & Analytical Sciences Rothamsted Research Harpenden UK; ^2^ Plant Sciences Rothamsted Research Harpenden UK; ^3^ The Earlham Institute Norwich UK; ^4^ Global Wheat Program, International Maize and Wheat Improvement Centre (CIMMYT) Texcoco Mexico

**Keywords:** enrichment capture sequencing, gene‐based association analysis, partitioning heritability per gene, signature of selection, trehalose phosphate phosphatase, trehalose phosphate synthase

## Abstract

Trehalose 6‐phosphate (T6P) signalling regulates carbon use and allocation and is a target to improve crop yields. However, the specific contributions of trehalose phosphate synthase (TPS) and trehalose phosphate phosphatase (TPP) genes to source‐ and sink‐related traits remain largely unknown. We used enrichment capture sequencing on TPS and TPP genes to estimate and partition the genetic variation of yield‐related traits in a spring wheat (*Triticum aestivum*) breeding panel specifically built to capture the diversity across the 75,000 CIMMYT wheat cultivar collection. Twelve phenotypes were correlated to variation in TPS and TPP genes including plant height and biomass (source), spikelets per spike, spike growth and grain filling traits (sink) which showed indications of both positive and negative gene selection. Individual genes explained proportions of heritability for biomass and grain‐related traits. Three *TPS1* homologues were particularly significant for trait variation. Epistatic interactions were found within and between the TPS and TPP gene families for both plant height and grain‐related traits. Gene‐based prediction improved predictive ability for grain weight when gene effects were combined with the whole‐genome markers. Our study has generated a wealth of information on natural variation of TPS and TPP genes related to yield potential which confirms the role for T6P in resource allocation and in affecting traits such as grain number and size confirming other studies which now opens up the possibility of harnessing natural genetic variation more widely to better understand the contribution of native genes to yield traits for incorporation into breeding programmes.

## Graphical abstract

The T6P signalling pathway is a central regulatory system of resource allocation and source‐sink interactions and is emerging as an important target in crops such as maize, rice, wheat and sorghum (Paul et al., 2018; Paul et al., 2020). Here, for the first time, we analysed comprehensive exome SNP information for TPS and TPP genes and dissected the genetic architecture of yield‐related traits in a spring wheat panel specially designed to represent the genetic diversity of 75,000 CIMMYT lines (Molero et al., 2019). The data showed significant relationships of TPS and TPP genes with agronomic traits with evidence of historical selection and identified opportunities for future selection of TPS and TPP genes and strategic crossing for yield improvement.

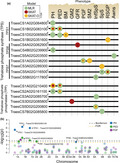

## 1 INTRODUCTION

The genetic improvement of wheat for increased yields is an urgent challenge. In bread wheat (*Triticum aestivum* L.), studies are increasingly focusing on the relationship between the supply of assimilates and the capacity to utilize carbohydrates, that is source and sink and their integration to increase genetic gains for yield (Reynolds et al., 2017). The trehalose pathway as a sugar signalling system consisting of trehalose phosphate synthase (TPS) and trehalose phosphate phosphatase (TPP) genes is emerging as a central regulator of both source‐ and sink‐ related traits. These traits encompass growth and development of the source including shoot leaf area, architecture and photosynthesis, and processes in sinks such as grain number and size (Fichtner et al., 2021; Paul et al., 2020b).

The trehalose pathway has an indispensable function in plants through the intermediate trehalose 6‐phosphate (T6P) as a signal of sucrose availability (Lunn et al., 2006; Schluepmann et al., 2003). T6P is an inhibitor of SnRK1 (Zhang et al., 2009), a member of the AMPK/SNF1 group of protein kinases. These kinases coordinate cellular and organismal responses to carbon and energy. Uniquely in plants T6P conveys information about carbon status to this central regulator. Through SnRK1, T6P de‐represses gene expression for carbon use in biosynthetic pathways and growth and development (Nunes et al., 2013; Zhang et al., 2009). On this basis, the pathway is a promising candidate for potential modification in crops to alter growth, development, architecture and the biosynthetic pathways that underpin the accumulation of yield‐determining end‐products such as starch (Paul et al., 2018). Grain‐related traits are already known to be regulated by this pathway. For example, overexpression of a TPP gene in maize led to improved grain set (grain numbers) and yield in the field (Nuccio et al., 2015). In bread wheat, a TPP gene was associated with grain weight (Zhang et al., 2017). TPP genes contribute to the difference in plant height and assimilate partitioning in sweet and grain sorghum (Li et al., 2019). The chemical intervention of T6P levels in wheat through the application of UV‐cleavable T6P precursors increased grain size in well‐watered conditions and enhanced vegetative growth recovery after drought stress (Griffiths et al., 2016). Consistent with a central function in the regulation of carbon and energy balance, Kretzschmar et al., (2015) showed that a TPP gene, *OsTPP7*, as the genetic determinant in qAG‐9–2, a major quantitative trait locus (QTL) for the promotion of anaerobic germination under flooding in rice. All these examples show the centrality of the pathway in determining yield processes and significantly both TPS and TPP genes are listed as having been modified during domestication in maize (Hufford et al., 2012), potato (Xu et al., 2017) and sugarcane (Hu et al., 2020) yet the association with traits and specific opportunities for further genetic enhancement of the pathway through selective breeding is not clear.

Recently, a spring wheat panel was specifically built to capture the genetic diversity of the 75,000 wheat cultivar collection to be available for the wheat community. The panel, known as the High Biomass Association Mapping Panel (HiBAP), consists of bread spring wheat lines constructed from elite high‐yielding material, pre‐breeding lines, landraces and synthetically derived lines selected for high yield and biomass. Marker‐trait associations have recently been published for this population (Molero et al., 2019). To complement the genome‐wide association studies, the genetic contribution of specific regulatory pathways to complex traits through variation inside genes is emerging as a new approach to understanding the role of key pathways and regulatory mechanisms to enable selection in crop breeding (Gardiner et al., 2018; Jordan et al., 2015; Uauy et al., 2017). Exome capture (enrichment) sequencing (Winfield et al., 2012) has been used for gene‐based association analysis (Neale & Sham, 2004), and screening signatures of selection in the whole genome in barley (Russell et al., 2016) and wild emmer (Avni et al., 2017), but selection signals in specific regulatory genes have not been performed in wheat. The use of genic variants for gene‐based prediction within regulatory pathways is showing great promise for application in breeding (Edwards et al., 2016; Zhang et al., 2020).

In this study, we used enrichment capture sequencing on the 25 TPS and 31 TPP genes found in wheat (Paul et al., 2018) for the dissection of the genetic architecture of 24 traits in the wheat HiBAP panel specifically developed to encompass genetic diversity across the 75,000 CIMMYT wheat collection (Molero et al., 2019). The overall aim was to better understand the extent to which variation in TPS and TPP genes was related to crop traits, evidence for selection that has already occurred, and evidence of ongoing selection and future selection possibilities. The hypothesis is that a central mechanism of sucrose resource allocation will already have been selected for crop improvement, but recent genetic and chemical interventions in crops (Paul et al., 2020b) indicate that further improvement and selection are most probable. Specific goals were to (i) apply single variant analysis and gene‐based approaches to maximize the detection of genetic associations, as both methods have different assumptions (univariate and multivariate distribution) about the genetic effects, (ii) evaluate the intragenic patterns of signatures of selection and epistatic interaction to find evidence of ongoing selection, (iii) estimate the genetic contribution of trehalose genes to the variation of complex traits across and within exotic‐derived and elite subpopulations, and (iv) explore various genomic prediction models using the whole genome and trehalose genes to predict complex traits. Our study has generated a wealth of information regarding links of TPS and TPP genes to yield traits, historical and ongoing selection which will serve to direct strategies of crossing and selection from a diverse CIMMYT genetic resource and for in‐depth mode of action studies to define the specific contribution of TPS and TPP genes to yield‐related traits.

## 2 MATERIALS AND METHODS

### 2.1 Enrichment capture sequencing and variant calling

Enrichment capture and bioinformatics analysis were carried out as per Joynson et al., (2021). Briefly, DNA was extracted from flag leaf material from each panel member. A combination of 10 leaves per plot was pooled before extraction using a standard CTAB method.

Sequences for 25 TPS and 31 TPP genes were taken from Paul et al., (2018). For TPSs these were annotated as *TPS1*, *TPS6*, *TPS7* and *TPS11* in accordance with the nearest *A*. *thaliana* TPS (Paul et al., 2018). For TPPs this was not possible due to greater genetic divergence in TPPs between the two species. Probe sequences (120 bp) were designed in an end‐to‐end format targeting gene bodies and 2000 bp upstream ensuring capture of each gene's promoter sequence. These were integrated into a 12 Mb capture probe set. Libraries were constructed using the TruSeq DNA library preparation kit (Illumina) and were sequenced on a NovaSeq6000. The 150 bp paired‐end sequences were mapped to the Refseq‐v1.0 reference sequence using BWA MEM version 0.7.13 73 with subsequent filtering carried out using SAMtools v1.4 and Picard Tools MarkDUplicates. Variants were called using bcftools and filtered using GATK (McKenna et al., 2010).

### 2.2 Mapping population and phenotypic traits

Mapping population and phenotypic data analyses have been described by Molero et al., (2019). Briefly, we used the HiBAP (High Biomass Association Panel) population which was specifically built to capture the genetic diversity across the 75,000 lines of the CIMMYT wheat collection through 149 wheat spring genotypes of the wheat pre‐breeding and breeding programme. This panel comprised two main subpopulations of 97 elite lines and 52 exotic derivatives (landraces, synthetic and introgression lines). The field trials were conducted in two consecutive growing seasons (2015/16 and 2016/17) under fully irrigated conditions situated in the Yaqui Valley, Mexico.

We used the means adjusted for spatial and temporal factors of 24 phenotypes: plant height (PH, cm), peduncle length (PED, cm), biomass at physiological maturity (BM, g/m^2^), harvest index (HI), yield (Yield, g/m^2^), thousand grain weight (TGW, g), grains per m^2^ (GM2), percentage of grain filling (PGF), grain filling rate (GFR, yield/grain filling duration, g/m^2^/day), spikes per m^2^ (SM2), grains per spike (GSP), grain weight per spike (GWSP, g), spikelets per spike (SpS, number), infertile spikelets per spike (InfSpS), spike length (Spike, cm), awn length (Awns, cm), rapid spike growth phase (RSGP, percentage), days to initiation of booting (DTInB), days to anthesis (DTA), days to maturity (DTM), thermal time to initiation of booting (TTInB), thermal time to anthesis (TTAnth), thermal time to maturity (TTPM) and thermal time to anthesis +7 days (TTA7H). For further detail on trait evaluation see Molero et al., (2019).

### 2.3 Variant filtering and annotation

Twenty‐one TPS and 27 TPP genes showed at least one variant and these were submitted to variant filtering (Table S2). For the gene‐based analysis, we applied two strategies of variant filtering using (a) MAF ≥ 0.01 as combining low and common variants in region‐based testing is likely to improve the statistical power of the models in detecting associations (Timpson et al., 2018), and (b) MAF ≥ 0.05. For the single‐point analysis, we only used markers with MAF ≥ 0.05. For the remaining genetic analyses, we used MAF ≥ 0.01 to capture more variation inside genes. Markers with call rate (CR) <95% were removed, and the remaining missing variants were imputed using Beagle 4.1 (Browning & Browning, 2016) within the *codeGeno* function from the Synbreed R package (Wimmer et al., 2012). The final genotypic matrix was composed of 749 (1% MAF) and 319 (5% MAF) variants. The Ensembl Plants (Bolser et al., 2016) variant effect predictor (VEP) tool (McLaren et al., 2016) was used to annotate variants (coding and non‐coding substitutions) and retrieve the functional impact scores of non‐synonymous mutations according to the Sorting Intolerant From Tolerant (SIFT) algorithm (Vaser et al., 2016).

### 2.4 Inference of population structure and genetic differentiation

We explored the gene ontology network (biological process) of the trehalose biosynthetic pathway using the Cytoscape ClueGo plug‐in (Bindea et al., 2009) inputting the wheat reference genome (IWGSC RefSeq v1.0 annotation) from Ensembl Plants. Additionally, we estimated the genetic diversity of exome variants by calculating the polymorphic information content (PIC) and MAF using the *popgen* function from snpReady R package (Granato et al., 2018).

We detected the genomic diversity structure of the population at the gene level. First, we applied a principal component (PC) analysis using the SNPRelate R package (*snpgdsPCA* function; Zheng et al., 2012). Second, we applied a discriminant analysis of principal components (DAPCs) using the adegenet R package (Jombart et al., 2010). The group clustering used was inferred by Molero et al., (2019). The contributions (loadings) of each gene variant were estimated using the *loadingplot* function. Finally, a neighbour‐joining tree (NJT) was generated based on the modified Euclidean distance using the ape R package (Paradis et al., 2004) and the pairwise genetic distance between populations (*F*_ST_) was calculated following Weir and Cockerham (1984) in the SNPRelate R package. The genome‐wide marker data (9267 variants remaining after quality control), generated using the 35 K Affymetrix Axiom® HD wheat SNP array (Allen et al., 2017), was used only for the DAPCs.

We estimated the level of linkage disequilibrium (LD) between and within trehalose genes using the square allele frequency correlation coefficient (*r^2^*) calculated for each pairwise combination in PLINK v.1.9 (Purcell et al., 2007). The LD decay curve was fitted by a non‐linear regression model (Marroni et al., 2011), obtained by fitting *r^2^* with distance using Hill and Weir expectation of *r^2^* between adjacent sites (Hill & Weir, 1988; Remington et al., 2001). Haplotype LD block was visualized in elite and exotic subgroups separately using the LDheatmap R package (Shin et al., 2006). Pairwise variant interactions between gene regions were tested by a linear regression analysis using PLINK ‐‐epistasis command. Regression coefficients (betas) were estimated for each interaction. The Bonferroni multiple testing was used to correct the epistatic significance threshold (0.05/N), where N is the number of interactions tested.

### 2.5 Single‐point scan and gene‐based mapping

Single variant association analysis was performed using a Mixed Linear Model (MLM) in GAPIT v3.0 R package (Lipka et al., 2012; Wang & Zhang, 2018) incorporating genomic kinship (K) matrix and the first three PCs (Q) to control for the confounding effects of cryptic relatedness and population structure (Yu et al., 2006). The default false discovery rate (FDR) (Benjamini & Hochberg, 1995) and Bonferroni multiple testing (Hochberg, 1988) were used to correct the genome‐wide significance thresholds (α = 0.05).

Following recommendations that region‐based tests have different assumptions about the genetic effects and weighting functions (Bomba et al., 2017; Lee et al., 2014; Nicolae, 2016), we measured the performance of gene mapping empirically using three approaches. First, we used a traditional multiple linear regression (MLR) model (Chapman & Whittaker, 2008) considering genotype effects as fixed. Second, we applied the SKAT model (Chen et al., 2013; Wu et al., 2011) assigning an Identity by State (IBS) kernel function. Third, we used the combination of burden test and SKAT named SKAT‐O (Lee et al., 2012, 2014). Both kernel‐based tests consider the genotype effects as random. Variance components were estimated using restricted maximum likelihood (REML). The weights were calculated using the standard probability density function of the beta distribution. For further detail on the model description see Svishcheva et al., (2019). We estimated the *P*‐values by using Kuonen's method (Kuonen, 1999) and considered the mode of inheritance as additive. The genomic relationship matrix (GRM) was calculated using the first formula proposed by VanRaden (2008), and the first three PCs were used as covariates in the models. Gene‐based mapping was performed using the *MLR* and *FFBSKAT* (*rho* was assigned for SKAT‐O test) functions in the FREGAT R package (Belonogova et al., 2016). Genes containing only one variant were removed from the analyses. We included all variant annotations (coding and non‐coding) in the tests (Neale & Sham, 2004) following suggestions that combining signals from multiple mutations in the same gene increases model statistical power (Sham & Purcell, 2014). We adjusted the *P*‐values for multiple comparisons to control for type I error at α = 0.05 using the traditional FDR and Bonferroni procedure (0.05/N, where N is the number of genes tested) using the *p*.*adjust* R function. Finally, quantile–quantile (Q‐Q) plots were used to verify the fitness of the model and plotted using the CMplot R package (https://github.com/YinLiLin/R‐CMplot).

### 2.6 Screening for signature of selection at the gene level

We evaluated the evidence of selection at the gene level by estimating the normalized ratio of non‐synonymous (missense, nonsense and splicing) substitutions per synonymous site (ω =* d*_N_/*d*_S_) using an optimized Poisson‐based model (*dNdScv*) in the dndscv R package (Martincorena et al., 2017). Briefly, this model accounts for variation in mutation rates, sequence context and full trinucleotide mutability. To estimate the mutation rate of a gene it uses a joint likelihood function combining local (synonymous substitutions in a gene) and global (negative binomial regression across genes) information to estimate the mutation rate of a gene. We used the *buildref* function to input the wheat reference genome (IWGSC RefSeq v1.0 annotation) from Ensembl Plants per chromosome. Global ω estimates across all genes were estimated per chromosome. A global *q*‐value ≤ 0.1 (without considering InDels) was used to identify statistically significant genes. A confidence interval (α = 0.95) was calculated per gene. Selection was measured as positive (ω > 1), negative (ω < 1) and neutral (ω = 1; Nielsen, 2005).

### 2.7 Partitioning heritability per gene and predictive models

We investigated distributions of population genetic parameters by estimating beta and effect size. First, we estimated the coefficient of regression (β) by fitting a single‐point association test (Q+K model) using the FREGAT R package. Briefly, beta is the absolute additive effect of the minor alleles on the phenotype in standard deviations (Park et al., 2011; Timpson et al., 2018). Second, we estimated the effect size, defined as the contribution of the variant to the genetic variance of the trait, following the equation: $EF=2\beta^{2}f\left(1‐f\right)$, where β measures the regression effect, and f denotes the minor allele frequency (Park et al., 2010, 2011).

We further investigated the genetic architecture of complex traits by partitioning the genetic variation of individual genes and gene families within and across elite and exotic subpopulations using the genomic‐relatedness‐based restricted maximum likelihood (GREML) approach (Yang et al., 2010) implemented in GCTA software v1.93.1beta (Yang et al., 2011). To estimate the proportion of the phenotypic variance explained (i.e. genomic heritability) per gene we fitted multiple GRM in the model, one contributed by the whole genome (35 K SNP Chip) and a second by a specific gene region. The proportion of heritability was estimated ignoring population structure (Table 1; Figure 5) and adjusting PCs as fixed covariates (Table S1). We reported the single gene heritability as $h_{l}^{2}=\sigma_{l}^{2}/\left(\sigma_{g}^{2}+\sigma_{l}^{2}+\sigma_{\varepsilon }^{2}\right)$ (see Methods S1). Additionally, we partitioned the variation of the gene family as $h_{\mathrm{TPS}}^{2}=\sigma_{\mathrm{TPS}}^{2}/\left(\sigma_{g}^{2}+\sigma_{\mathrm{TPS}}^{2}+\sigma_{\mathrm{TPP}}^{2}+\sigma_{\varepsilon }^{2}\right)$ (see Methods S2). $\sigma_{l}^{2}$, $\sigma_{g}^{2}$, $\sigma_{\mathrm{TPS}}^{2}$, $\sigma_{\mathrm{TPP}}^{2}$ and $\sigma_{\varepsilon }^{2}$ are the local gene, global whole genomic, TPS and TPP gene family, and residual variances, respectively.

**TABLE 1:** Summary of trehalose phosphate synthase (TPS) and trehalose phosphate phosphatase (TPP) genes significantly associated with yield‐related traits from the gene‐based analysis in the wheat HiBAP panel

Gene family	Gene class	Gene ID^a^	No. of variants^b^	*Ω* ^c^	Affected trait^d^	Proportion of heritability^e^		
						Elite	Exotic	All
Trehalose phosphate synthase	*TPS1*	TraesCS1A02G064800^a^	136	0^(0−0.3)^	PH	0.48 ± 0.19	0.00 ± 0.07	0.26 ± 0.16
	*TPS1*	TraesCS1B02G083100^a^	89	1.16^(0.2−3)^	PH*	0.70 ± 0.13^*^	0.00 ± 0.05	0.62 ± 0.14^*^
					PED*	–	–	–
	*TPS1*	TraesCS1D02G065600^a^	67	1.91^(0.3−5)^	PH	0.56 ± 0.16^*^	0.00 ± 0.12	0.49 ± 0.14^*^
					PED	0.26 ± 0.19	0.00 ± 0.03	0.10 ± 0.10
					BM	0.14 ± 0.14	0.01 ± 0.06	0.12 ± 0.10
	*TPS1*	TraesCS1B02G351600	10	0.26^(0−1)^	RSGP	0.01 ± 0.04	0.24 ± 0.23	0.13 ± 0.13
	*TPS6*	TraesCS4A02G062900^b^	8	0	GFR	0.05 ± 0.06	0.00 ± 0.09	0.02 ± 0.03
	*TPS6*	TraesCS4B02G239900^b^	5	–	PH	0.00 ± 0.09	–	0.58 ± 0.34
	*TPS6*	TraesCS5A02G203500	14	0.50^(0.2−2)^	Awns	0.91 ± 0.07	–	0.86 ± 0.11
	*TPS7*	TraesCS1A02G338200	26	0.18^(0−0.8)**^	PGF*	0.85 ± 0.11^*^	0.01 ± 0.05	0.66 ± 0.21^*^
					SM2	0.35 ± 0.25	–	0.34 ± 0.23
	*TPS7*	TraesCS3A02G289300	19	0.70^(0.1−2)^	SpS	0.00 ± 0.02	0.00 ± 0.09	0.00 ± 0.02
	*TPS7*	TraesCS5A02G116500^c^	2	0^(0−1.4)^	SpS	0.05 ± 0.07	–	0.05 ± 0.06
					RSGP	0.03 ± 0.05	–	0.04 ± 0.05
	*TPS7*	TraesCS5B02G117800^c^	4	–	InfSpS*	0.00 ± 0.03	0.07 ± 0.12	0.00 ± 0.03
Trehalose phosphate phosphatase	–	TraesCS1A02G210400	4	1.20^(0.2−3)^	PH*	0.02 ± 0.04	0.00 ± 0.09	0.02 ± 0.04
					PED*	0.09 ± 0.12	0.00 ± 0.19	0.04 ± 0.06
	–	TraesCS2A02G161100	14	0.50^(0.2−2)^	PH	0.00 ± 0.03	–	0.00 ± 0.02
					PED	0.00 ± 0.02	–	0.00 ± 0.01
	–	TraesCS3A02G085700^a^	19	0^(0−1.2)^	InfSpS*	0.00 ± 0.04	0.00 ± 0.06	0.00 ± 0.06
	–	TraesCS3D02G085800^a^	65	–	GM2*	–	0.58 ± 0.22^*^	0.15 ± 0.13
	–	TraesCS5D02G200800	2	4^(0.2−17)^	InfSpS	0.04 ± 0.06	0.13 ± 0.15	0.07 ± 0.08
	–	TraesCS7B02G085800	5	–	SpS*	0.00 ± 0.02	0.05 ± 0.12	0.01 ± 0.03

^a^ Wheat gene ID at EnsemblPlants (IWGSC RefSeq v1.0 annotation). Same letter indicates homoelogues genes.

^b^ Number of variants inside the gene after applying for quality control using 1% MAF.

^c^ Ratio (ω = *d*_N_/*d*_S_) of the number of non‐synonymous mutations (*d*_N_) to the number of synonymous mutations (*d*_S_). Error depict 95% confidence interval (CI). **Genes detected at α < 0.1 (qglobal, Benjamini–Hochberg adjustment). Values not shown represent that the model did not converge.

^d^ Traits are plant height (PH, cm), peduncle length (PED, cm), final biomass (BM, g/m^2^), grains per m^2^ (GM2), grain filling rate (GFR, yield/grain filling duration, g/m^2^/day), percentage of grain filling (PGF), spikes per m^2^ (SM2), infertile spikelets per spike (InfSpS, number), spikelets per spike (SpS, number), rapid spike growth phase percentage (RSGP) and awn length (Awns, cm). *Significant trait detected using MAF ≥ 0.01 and MAF≥0.05 by at least one gene‐based model.

^e^ Proportion of heritability per single gene from elite, exotic derivatives and the complete panel. Values are mean ± standard errors. Values not shown represent that the model did not converge. *Significant genes by Bonferroni correction at α = 0.05 detected using the regional heritability mapping (RHM).

We used the additive genomic best linear unbiased prediction (GBLUP) model controlling for population structure (Lyra et al., 2018) to compare the predictive ability of four gene‐based approaches (see Methods S3). Prediction of the phenotypes was performed by using the (i) genome‐wide marker (35 K SNP Chip) effects, (ii) TPS and (iii) TPP gene family effects, and (iv) combining the whole‐genome variation with the effects of the TPS and TPP gene families.

## 3 RESULTS

### 3.1 Exome sequences from trehalose pathway genes revealed substantial within‐group variation in elite germplasm

**FIGURE 1:**
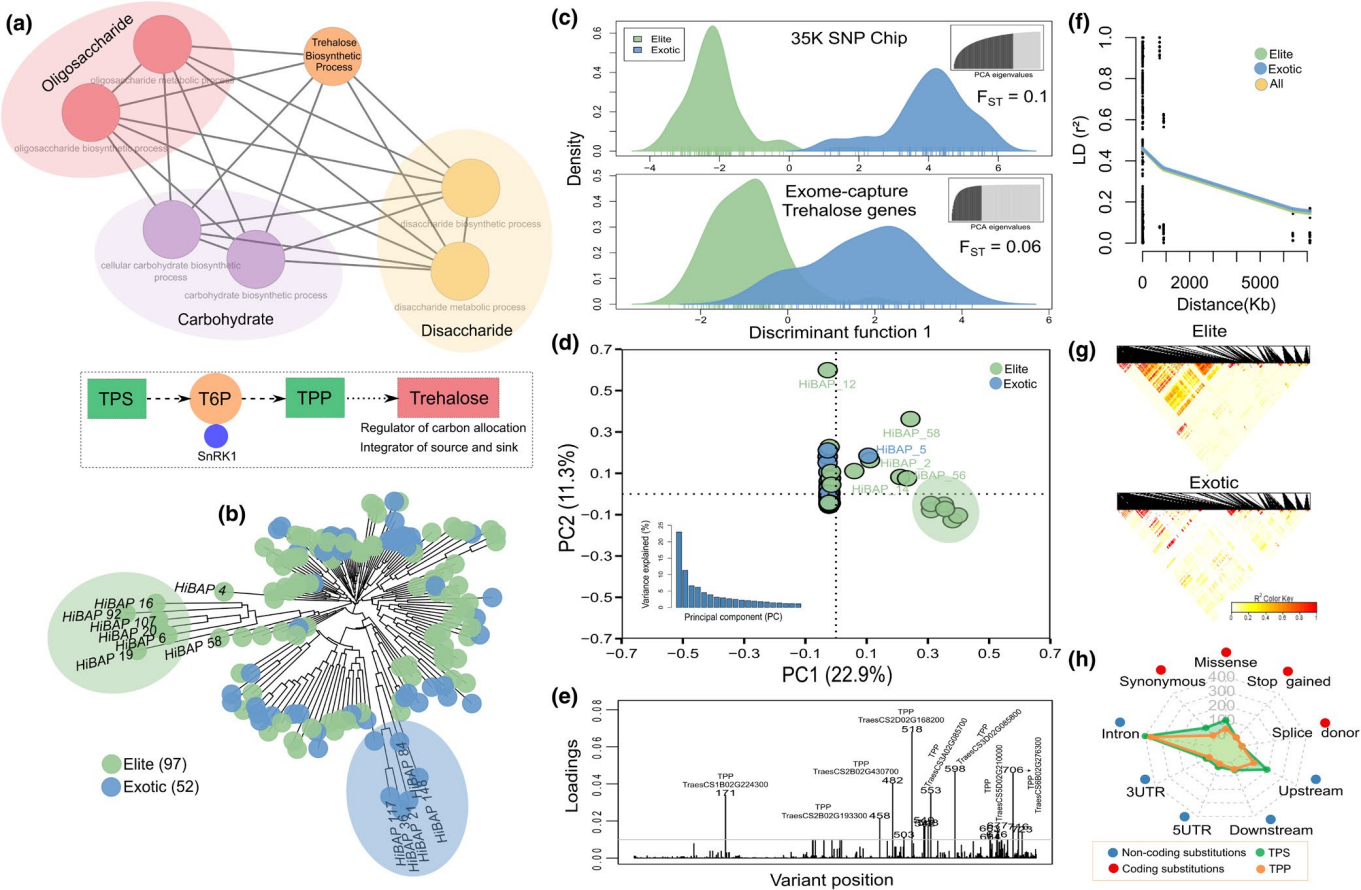
Population structure analysis using the exome capture data in the wheat HiBAP panel. (a) Gene ontology network and summary of the trehalose biosynthetic pathway. The same colour nodes represent similar biological processes. Trehalose phosphate synthase (TPS), trehalose 6‐phosphate (T6P) and trehalose phosphate phosphatase (TPP). (b) Neighbour‐joining tree (NJT) based on Euclidean distance where each colour represents a group. Group clustering was determined by Molero et al., (2019). (c) Density of individuals from a single discriminant function using the 35 K SNP Chip and exome capture data. Dark grey colour on the top right is the number of principal components (PC) retained for the discriminant analysis (DA). *F*_ST_ values are shown inside the plots. (d) First two PCs using exome data coloured by groups. Bottom left plot represents the variance explained by the first twenty PCs. (e) Contributions (loadings) of each gene variant to the DA function. (f) Pattern of linkage disequilibrium (LD) decay among all pairs of genetic variants for the complete set of individuals (all), elite and exotic materials. Values reported are the average squared correlations (*r* ^2^) across all genes. (g) LD heatmap of the gene variants for elite and exotic subgroups. The colour gradient scale represents the range of *r* ^2^ values. Black represents the highest estimates of LD. (h) Radar plot showing the distribution of Variant Effect Predictor (VEP) consequences (five non‐coding and four coding substitutions) for the trehalose phosphate synthase (TPS) and trehalose phosphate phosphatase (TPP) gene family

We generated the gene ontology network and a framework of the trehalose biosynthetic pathway (Figure 1a). Genome‐wide (*F*_ST_ = 0.1) and exome profiles (*F*_ST_ = 0.06) revealed mild genetic structuring between groups and a substantial within‐group variation in elite lines, suggesting that these genotypes have gene‐specific mutations (Figure 1b–d). Interestingly, the most contributing alleles to classify the groups were predominantly TPP variants (Figure 1e). We found that gene‐wide LD decayed relatively slowly in the panel, implying that many genetic variants between genes remained correlated, also suggesting reduced recombination rates due to artificial selection (Figure 1f). Elite lines revealed a larger LD block between genes compared to exotic materials, indicating fewer recombination events, most likely due to bottlenecks created by the development of elite inbred lines (Figure 1g).

### 3.2 Gene‐based scanning detected multiple trehalose pathway genes associated with key agronomic traits

Exome capture data showed that the number of variants in TPS and TPP sequences varied greatly among genes from 1–173 in 21 TPS and 27 TPP homologues (Tables S1–S2), and most of them were predicted as non‐coding substitutions (e.g. introns and upstream) whereas, in exonic regions, missense (non‐synonymous) substitution was the most prevalent annotation (Figure 1h; Tables S1–S2).

**FIGURE 2:**
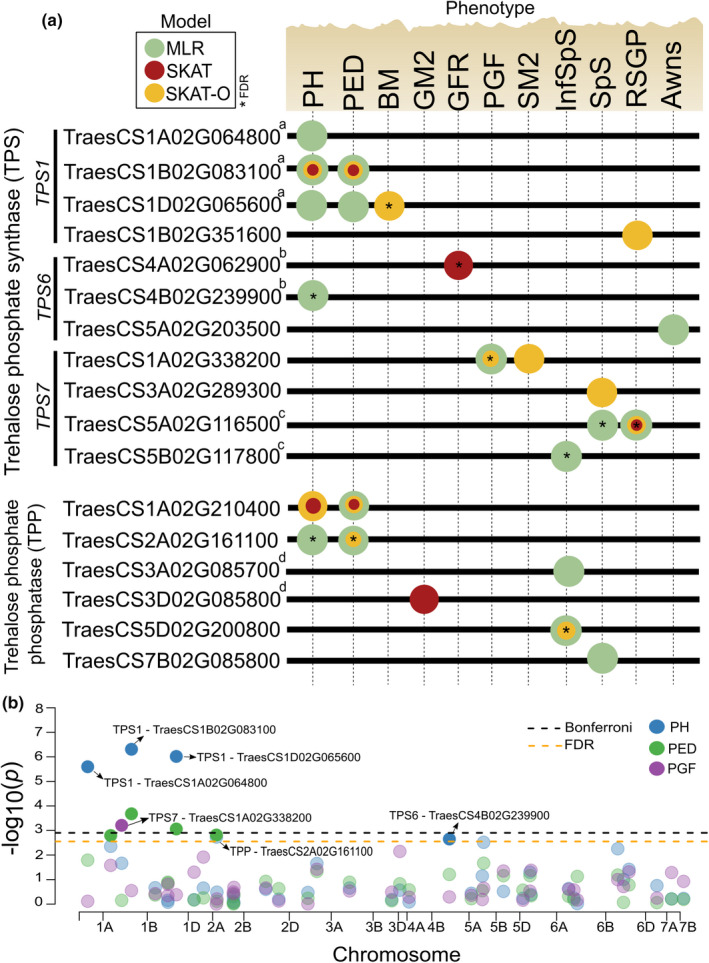
Summary of the gene‐based association analysis in the wheat HiBAP panel. (a) Trehalose phosphate synthase (TPS) and trehalose phosphate phosphatase (TPP) genes are shown in the left panel. Same letter indicates homoelogues genes. Circle forms encoded by different colours represent genes detected by sequence kernel association test (SKAT), optimized SKAT (SKAT‐O) and multiple linear regression (MLR) models. Gene‐trait association detected using minor allele frequency (MAF) ≥ 0.01. Significance level used is Bonferroni correction (no asterisk, α = 0.05) and False Discovery Rate (with asterisk, α = 0.05). Circles overlapping each other represent multiple models detecting the same gene. Only genes and traits on which significant associations were detected are shown in the figure. ^a−d^Same letter right to the gene ID indicates homoelogues genes. (b) Manhattan plot from the gene‐based mapping. Results shown are from the MLR model. The *x*‐axis shows genomic position (chromosomes 1A‐7B), and the *y*‐axis shows statistical significance [–log10(*P*)]. Dotted line indicates significance level for Bonferroni correction and False Discovery Rate (α = 0.05). Each dot represents a gene coloured by phenotype. Significant gene names are shown by black arrows. Traits are plant height (PH, cm), peduncle length (PED, cm), final biomass (BM, g/m^2^), grains per m^2^ (GM2), grain filling rate (GFR, yield/grain filling duration, g/m^2^/day), percentage of grain filling (PGF), spikes per m^2^ (SM2), infertile spikelets per spike (InfSpS, number), spikelets per spike (SpS, number), rapid spike growth phase percentage (RSGP) and awn length (Awns, cm)

From the single‐point scans, we detected three point mutations in the TPP gene TraesCS1A02G210400 linked with peduncle length and one variant in *TPS7* (TraesCS5B02G117800) associated with infertile spikelets per spike (SpS) (Table S3). Interestingly, all significant signals identified fell in non‐coding regions. The gene‐based mapping detected more signals than single variant analysis, identifying a total of 11 TPS and six TPP genes associated with 11 phenotypes using MAF ≥ 1% (Table 1; Figure 2; Figure S1), and seven genes linked to six traits using MAF ≥ 5% (Table 1). Plant height had the highest number of significant associations (i.e. four TPS and two TPP) followed by peduncle length. There were also effects on grain traits related to spikelet fertility such as number of spikelets per spike, grains and spikes per m^2^, and grain filling duration. There were small differences in the relative performances of the region‐based models, but we observed a slight advantage of the multiple linear regression approach for gene discovery showing good model fits (Figure 2; Figure S1).

### 3.3 Trehalose pathway genes revealed positive epistatic interactions, pleiotropy and distinct intragenic linkage disequilibrium patterns

**FIGURE 3:**
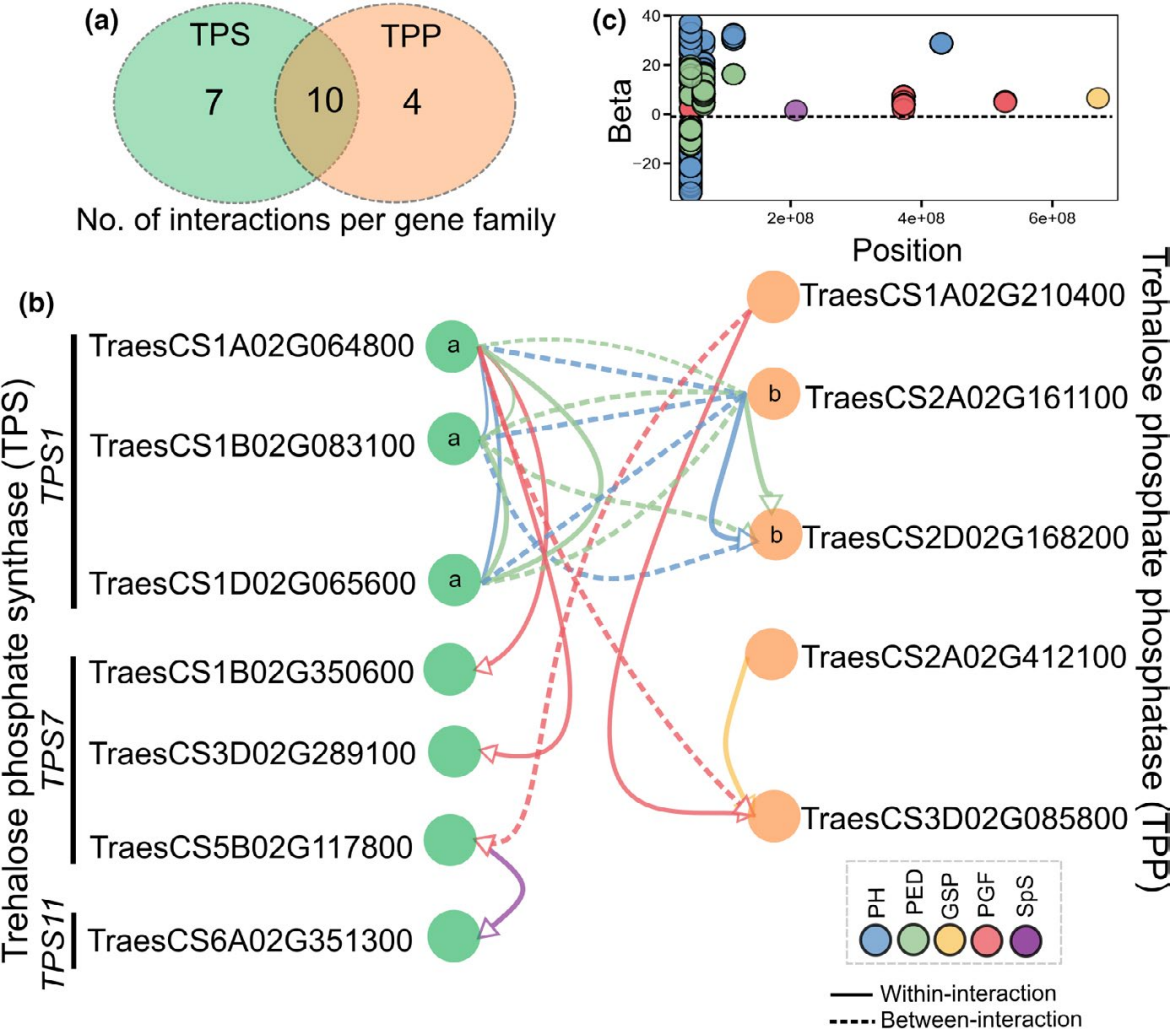
Epistatic interaction of polygenic traits across trehalose family genes in the wheat HiBAP panel. (a) Venn diagram shows the unique and shared number of gene interactions per family. (b) Significant SNP‐SNP interaction within‐ (solid arrow) and between‐ (dotted arrow) trehalose phosphate synthase (TPS) and trehalose phosphate phosphatase (TPP) gene families. Arrows encoded by different colours represent phenotypes in which interactions between gene variants were found in at least one occasion. Significance level used was Bonferroni correction (α = 0.05). ^a−b^Same letter inside circle indicates homoelogues genes. Only genes and traits on which significant connections were detected are shown in the figure. (c) Distribution of regression coefficients (betas) of the interaction against position (bp) of one variant for five phenotypes. Traits are plant height (PH, cm), peduncle length (PED, cm), grains per spike (GSP, number), percentage of grain filling (PGF) and spikelets per spike (SpS, number)

We identified significant epistatic interactions within and between trehalose pathway genes associated with five yield‐related traits, particularly for plant height and peduncle length, but also grains per spike, percentage of grain filling and spikelets per spike (Figure 3a–c; Table S1). A large fraction of the interactions was positively associated with the traits (positive betas, Figure 3c). Accordingly, we found connectivity between coding genetic variants, for example missense (*TPS1* on chromosome 1A) and synonymous variants (TPP on chromosome 3D) positively interacting with each other to enhance the percentage of grain filling. Furthermore, our results also showed that six genes affected multiple distinct phenotypic traits (i.e. pleiotropic effects), particularly the *TPS1* on chromosome 1D (TraesCS1D02G065600, Figure 2a).

By evaluating the extent of intragenic LD, we identified substantial variation among TPS and TPP genes (Figure S2). For instance, the LD in homologues followed distinct patterns with some persisting across longer distances (e.g. TraesCS1A02G064800) while others increased with physical distance (TraesCS1B02G083100) or decayed within 1000 base pairs (TraesCS1D02G065600) implying differences in associations of TPS and TPP genes with neighbouring alleles.

### 3.4 A large fraction of trehalose pathway genes are under positive and negative selection

**FIGURE 4:**
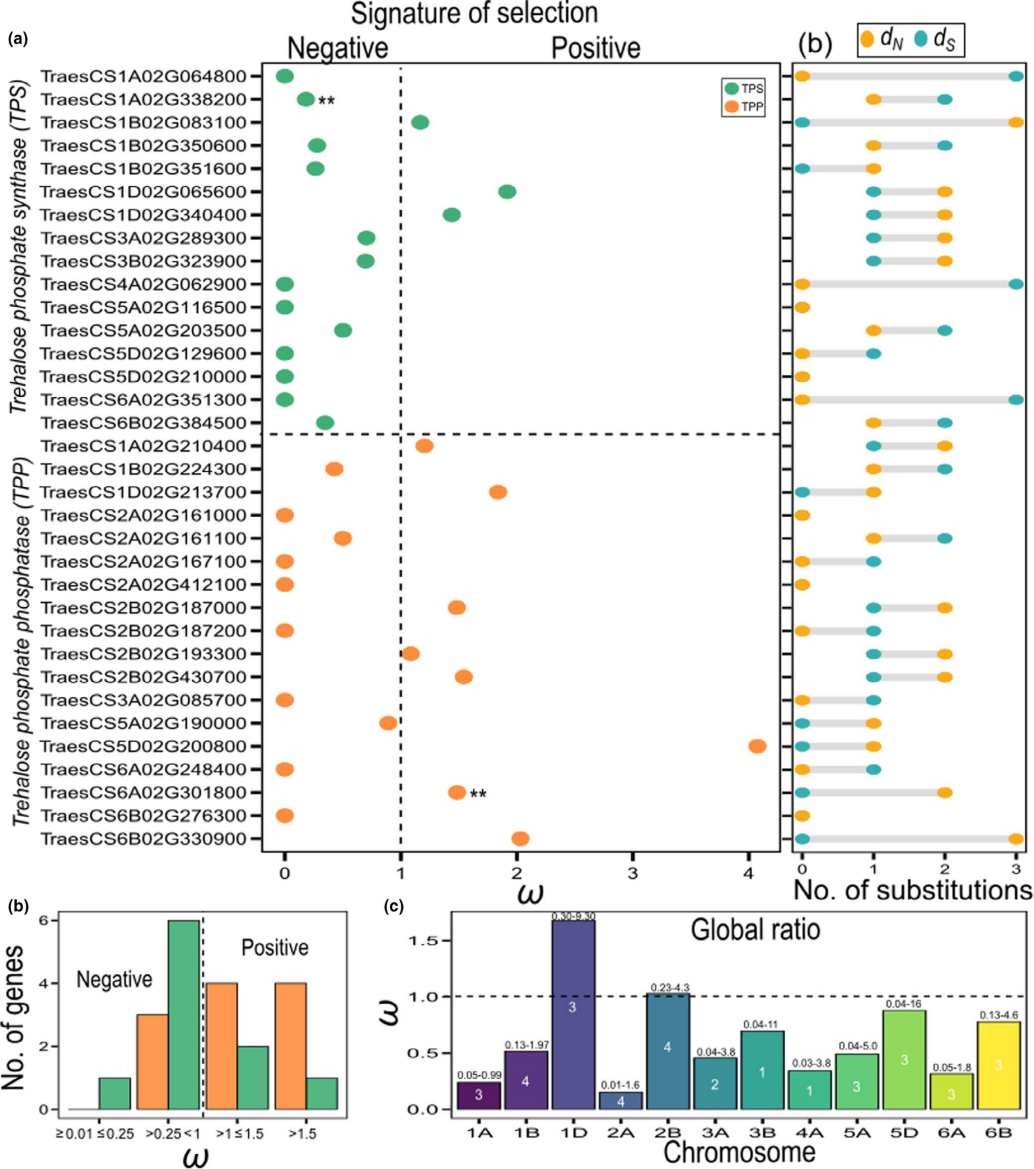
Inference of signature of selection across two trehalose family genes in the wheat HiBAP panel. (a) Gene‐wide ratio (ω) showing evidence of negative and positive selection. Only genes on which a ratio was estimated are shown in the figure. (b) Total number of non‐synonymous (*d*_N_) and synonymous (*d*_S_) substitutions per gene using the *dNdScv* method. **Genes detected at α ≤ 0.1 (qglobal). Trehalose phosphate synthase (TPS) and trehalose phosphate phosphatase (TPP) genes are shown in the panel. (c) Number of genes under different levels of positive and negative selection based on the ω ratio distribution. (d) Global ω estimates across all genes per chromosome. Values above bar plots indicate 95% confidence interval (CI). The number of genes (*n*) in each sub‐genome is given inside the plot

We measured the strength and mode of natural selection acting on regulatory regions via the *d*_N_/*d*_S_ ratio using a trinucleotide substitution model (Figure 4; Table 1). Nearly one‐half of the TPS and TPP genes showed strong indications of negative and positive selection indicating that purifying (negative) and diversifying (positive) selection are acting on them (Figure 4a). We observed high values (*ω *> 1, positive selection) of global information (i.e. variation of the mutation rate across genes in each sub‐genome) for the chromosomes 1D and 2B (Figure 4d). Interestingly, several genes found to be under positive selection were also associated with a specific phenotype, for example *TPS1* on chromosomes B and D (plant height and final biomass) (Figure 2; Figure 4) and a TPP gene TraesCS1A02G210400 (plant height and peduncle length). Some TPP genes showing positive selection were not linked to traits measured in our study (Figure 4a).

### 3.5 Partitioning the total genetic variance of trehalose genes revealed substantial contributions of the pathway to the phenotypic variance

**FIGURE 5:**
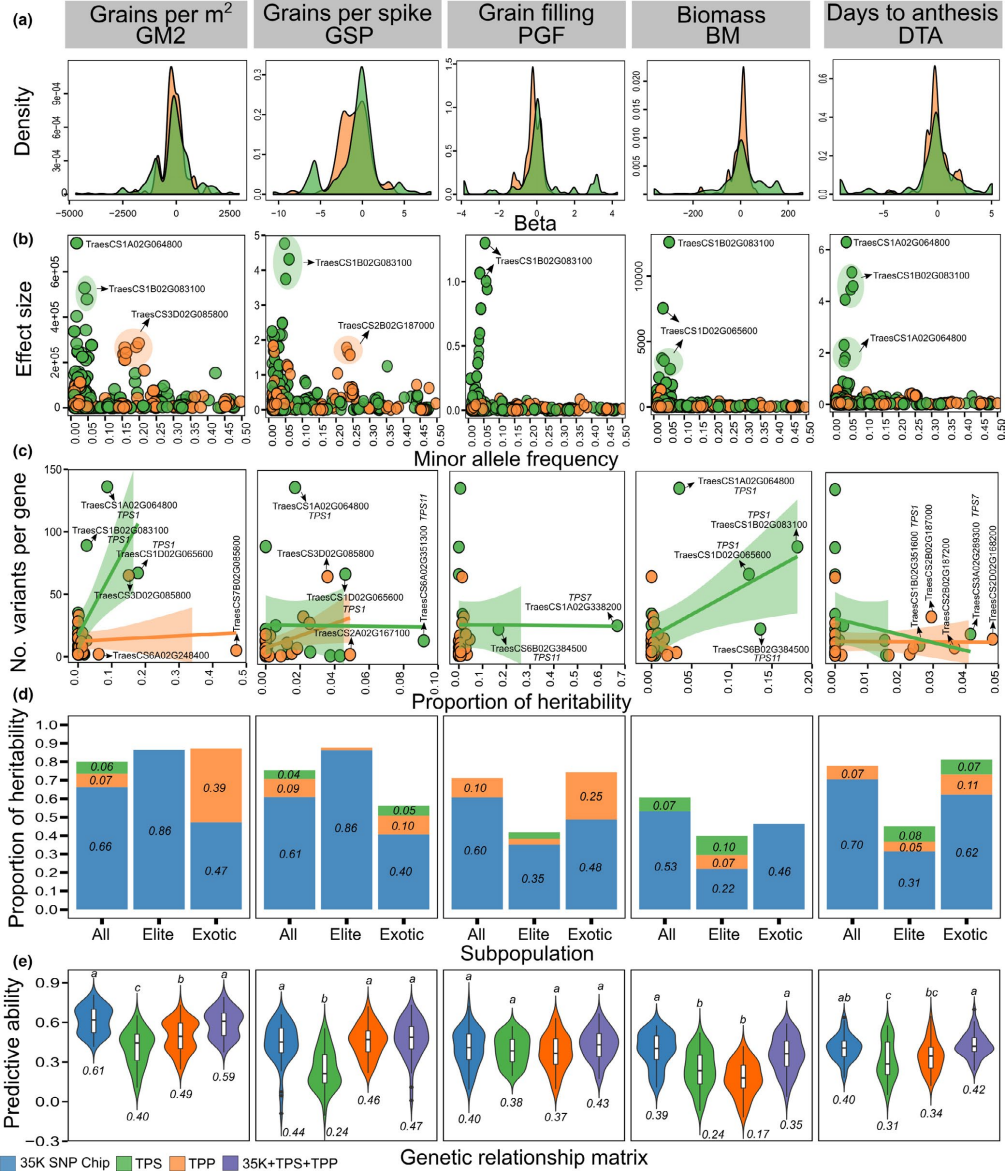
Genetic architecture of complex traits using exome capture data of trehalose genes in the wheat HiBAP panel. Gene families are trehalose phosphate synthase (TPS) and trehalose phosphate phosphatase (TPP). (a) Estimate of the density distribution of regression coefficients (betas). (b) Association between effect size (ES) and minor allele frequency (MAF). (c) Association between the number of variants and the proportion of heritability per gene. Some genes are shown by black arrows. (d) Proportion of heritability in the complete set, elite and exotic subgroups. (e) Predictive ability based on genome‐wide markers, single gene family and combined effects. Numbers inside plots represent the mean from 50 random cross‐validations. Different letters above violin plots indicate significant differences at α = 0.05 from Tukey's test. Genome‐wide markers represent the 35 K Affymetrix Axiom® HD wheat array. Traits are grains per m^2^ (GM2), grains per spike (GSP, number), percentage of grain filling (PGF), final biomass (BM, g/m^2^) and days to anthesis (DTA, days)

By quantifying the contribution of each variant to the genetic variance of the trait, we observed contrasting patterns of the beta densities, clearly showing differences in the peak and distribution across families (Figure 5a). Additionally, most of the variants with large effect sizes were found at low frequencies, following a decay curve shape (Figure 5b, Table S1).

We assessed the genetic architecture of complex traits by breaking down the total variance into single gene and gene families, thereby estimating the contribution of each component to the phenotypic variation (Figure 5c,d; Table 1; Table S1). The proportion of heritability per gene varied considerably between gene families (e.g. explaining up to 18% of the variance for biomass) (Figure 5c). Likewise, we showed that *TPS1* homologues explained a high fraction of heritability (with some significant regions) for thousand grain weight, grains per m^2^, plant height, final biomass and harvest index (Table 1; Table S1). We further observed a meaningful amount of the variance explained by the TPS family in the complete set (e.g. 0.13 of the heritability for grains per spike; Figure 5d). Intriguingly, a pronounced contribution (e.g. 0.02–0.41 of the heritability) of TPP genes was evidenced in the exotic germplasm, particularly for sink traits, for example grains per m^2^, grains per spike and percentage of grain filling.

Under the expectation that well‐known regulatory pathways could be used in gene‐based prediction, we identified that complex phenotypes were moderately predicted using only single‐family effects (e.g. 0.09–0.47 for TPS and 0.03–0.49 for TPP) (Figure 5e; Table S1). When we included gene effects simultaneously with whole‐genome markers, we observed gains of predictive ability for grain weight per spike (i.e. significant increase of 6% compared to the traditional model).

## 4 DISCUSSION

The T6P signalling pathway is a central regulatory system of resource allocation and source‐sink interactions and is emerging as an important target in crops such as maize, rice, wheat and sorghum (Paul et al., 2018, 2020a). Here for the first time we analysed comprehensive exome SNP information for TPS and TPP genes and dissected the genetic architecture of yield‐related traits in a spring wheat panel specially designed to represent the genetic diversity of 75,000 CIMMYT lines (Molero et al., 2019). We showed significant relationships of TPS and TPP genes with twelve agronomic traits with evidence of historical and ongoing selection and identified opportunities for future selection of TPS and TPP genes and potential epistatic interactions between TPS and TPP genes for yield improvement.

### 4.1 Gene‐based scanning detected multiple trehalose pathway genes associated with key agronomic traits

Previous genome‐wide and exome studies of complex traits suggested that both coding and non‐coding variants tend to contribute to the phenotypic variance (Li et al., 2016; Visscher et al., 2017). Consistent with this expectation, we identified exclusively non‐coding regions associated with the phenotypes (Table S3). Moreover, we empirically confirmed the argument that gene‐based mapping would have greater statistical power than conventional single analysis (Li & Leal, 2008) as fewer signals were detected using the latter approach. We also observed that multiple linear regression outperformed the methods with random‐effects (Figure 2a) showing a relatively good model fit (see QQ plots in Figure S1). Exome capture has proven in this study to be a very viable means to narrow down the relative importance of core network genes (Kiezun et al., 2012) and represents a good paradigm for such an approach for other regulatory pathways.

### 4.2 Trehalose biosynthetic genes revealed positive epistatic interactions, pleiotropy and distinct intragenic linkage disequilibrium pattern

Under the assumption that epistasis is relatively common in central genetic networks of highly polygenic traits (Mackay, 2014) we provided evidence of connectivity between variants from trehalose pathway genes, particularly for a set of complementary genes on the A, B and D genomes (Figure 3). For instance, we observed that the *TPS1* homologues interacted considerably with TPP homologues on chromosomes 2A and 2D to affect plant height and peduncle length. This is entirely to be expected because both TPS and TPP genes are likely to coordinate regulation of T6P levels and hence combine to impact traits. Trehalose pathway gene interactions have been reported in nematodes and yeast (Apweiler et al., 2012; Kormish & McGhee, 2005). Our results also revealed pleiotropic effects (Figure 2; Table 1) likely because T6P elicits changes in whole plant carbon allocation to affect more than one trait together (Paul et al., 2018, 2020a) confirmed in transgenic studies (e.g. large changes in vegetative architecture and relationships with sucrose content) (Goddijn & van Dun, 1999; Lunn et al., 2014; Romero et al., 1997).

Contrasting patterns of gene LD have been reported across a range of studies in maize (Ching et al., 2002; Remington et al., 2001), barley (Caldwell et al., 2006), rice (Mather et al., 2007) and rye (Li et al., 2011), but the investigation of wheat genes remain largely unexplored (Sela et al., 2011). By evaluating a large set of exome regions (Figure S2) we identified high levels of intragenic LD (persisting and increasing across longer distances), possibly as a result of the reduced recombination rates due to strong artificial selection of associated alleles (Palaisa et al., 2003; Remington et al., 2001). We also observed a few occasions where LD decayed rapidly (i.e. *TPS1*‐TraesCS1D02G065600 and TPP‐TraesCS2D02G168200) reflecting the impact of local recombination, meaning that such genes could have been selected quickly during the breeding process.

### 4.3 A large fraction of trehalose pathway genes are under positive and negative selection

In the screens for signatures of selection, we identified a similar proportion of genes under positive and negative selection (Figure 4). Two *TPS1* homologues associated with plant height, peduncle length and biomass showed evidence of positive selection indicating they underwent breeding selection. Interestingly Li et al., (2019) found T6P levels associated with plant height and biomass in sweet and grain sorghum. Additionally, two *TPS7* genes on chromosomes 3A and 3B that are under negative selection in our study were identified as associated with domestication improvement in the closest genes in maize (Hufford et al., 2012; Paul et al., 2018). This implies that these genes may already have been selected and that further selection for yield is not being tolerated. Interestingly, a large proportion of the positive selection was attributed to the TPP family, indicating that most of the non‐synonymous substitutions in these genes might be essentially driver mutations, that is providing a selective advantage (Pon & Marra, 2015). TPP genes showed positive selection for traits such as percentage of grain filling (Figure 4). In contrast, for several TPS genes traits such as grains per m^2^, percentage grain filling, grain filling rate, spikelets per spike, grains per spike and final biomass showed negative selection, meaning that most of the mutations were removed by negative selection during crop breeding (Casillas & Barbadilla, 2017; Vitti et al., 2013). For instance, a significant ratio of *ω*~0.18 indicates that at least ~82% of missense mutations have been removed by negative selection.

### 4.4 Trehalose pathway genes revealed substantial contributions to the quantitative genetic variation of source‐ and sink‐related traits

We found that trehalose pathway genes contributed to proportions of heritability for specific traits, particularly for grain number and grain filling traits, biomass and days to anthesis (Figure 5c; Table S1), suggesting an important impact of this regulatory pathway on yield traits consistent with other studies (Paul et al., 2018). Comparing this result to those from the gene‐based testing (Figure 2), we found that the latter yielded fewer associations (e.g. the *TPS1* gene on chromosome 1D is linked to three traits but explained some variance to twelve traits). Additionally, a *TPS1* gene known to regulate flowering time in Arabidopsis (Wahl et al., 2013) explained around 2% of the heritability in our panel (Figure 5c). In a recent study, a TPP gene on chromosome 6A was associated with thousand grain weight in bread wheat and successfully cloned (Zhang et al., 2017). Similarly, we observed about 3% of heritability fraction in the same TPP gene associated with this particular trait.

We also estimated the relative contribution of each gene family to the phenotypic variance of subpopulations (Figure 5d; Table S1). As expected from the quantitative genetics’ theory (Barton et al., 2017) a larger proportion of heritability was captured by genome‐wide markers. Additionally, we observed contrasting contributions of the trehalose pathway family to elite and exotic materials. Firstly, both TPS and TPP gene families showed very little contributions to exotic germplasm for biomass, plant height and harvest index, but their proportion increased considerably in elite materials (up to 20% higher), suggesting that the selection process impacted carbon allocation and source and sink balance through the trehalose pathway. *TPS1* homologues explained a high fraction of heritability in elite materials, particularly for plant height (Table 1). The TPP family explained a high fraction of heritability in exotic materials for grain‐related traits (Figure 5; Table S1) and not so much for elite lines. TPP genes in exotic derivatives might contain more favourable alleles impacting grain‐related traits. This also suggests potential for inclusion of genetic variation in TPPs for these traits from exotic germplasm into breeding crosses to increase yield via these traits. Secondly, both gene families showed a high contribution to elite and exotic subpopulations for days to anthesis, suggesting that trehalose genes had little impact on flowering during the selection process in our panel, but suggest the pathway as a whole contributes substantially to flowering time, which was fixed early on in the breeding process. Similar patterns of genetic variation changes through selection have been reported across a range of complex traits (Briggs & Goldman, 2006; Raquin et al., 2008).

Our findings on predicting complex traits using trehalose gene variants have several important implications for designing strategic crosses aiming to improve source‐sink balance in wheat. As expected, predicting phenotypes by using single‐gene families captured enough information to represent the kinship supporting other studies that also show a clear advantage of gene‐based prediction compared to genome‐wide markers (Zhang et al., 2020). Our second strategy was combining whole‐genome marker effects with gene variants, and we observed that predictive ability was significantly improved only for grain weight per spike (Table S1). Genome‐wide marker effects are most likely capturing the variation from other core genes, and possibly the information of both types of kinship would be redundant, consequently not effectively contributing to improving prediction (Lyra et al., 2019). Incorporating gene effects considerably increased predictive ability for grain weight, suggesting that the associated regulatory pathway highly impacted the grain weight phenotype (see the proportion of heritability per single gene and subpopulation), thus adding extra information to the model.

### 4.5 Potential implications of using the trehalose pathway gene in wheat strategic crossing

Our hypothesis that the T6P pathway is in the middle of a selection process in wheat is supported by this study. Contribution of the pathway to the quantitative genetic variation of yield‐related traits enables the prioritization of target genes (*TPS1*, *TPS7*, *TPS11* homologues and several TPPs) (Table 1). Selection of genes within the pathway appears to be ongoing and positive for *TPS1* genes and several TPP genes, and already has had a significant impact on harvest index, final biomass, plant height and flowering time. It has already been demonstrated in transgenics, crossing and chemical intervention studies that perturbation of T6P has a large effect on the traits affected in the wheat study presented here (Paul et al., 2020b). However, it has not been possible before to relate traits to specific native genes in wheat in a comprehensive manner or show previous and ongoing selection and gene interactions in wheat. Hence the work contributes to targeting native genes for yield in specific ways that were not possible before. This is an important aspect in the improvement of food security through breeding or gene‐editing and yield improvements could be achieved through further selection.

We propose that the trehalose gene family will support designing strategic crossing and pre‐breeding in various ways. First, we identified several promising genes that in conjunction with gene‐editing techniques could be used to study their more exact role in source/sink pathways, for example *TPS1*, *TPS7* and *TPS11* homologues to elucidate their mode of action within the T6P pathway mechanism. Second, TPP genes in exotic‐derived material could be reintroduced to enhance grain‐related traits, for example grains per m^2^. Third, there were strong epistatic interactions between genes that could enable gene combinations to be considered, for example for *TPS1* and TPP genes for percentage of grain filling. Fourth, predicting wheat phenotypes by combining whole‐genome marker effects with trehalose pathway gene effects has the potential to be a viable predictive model, helping breeding programmes to design strategic crosses. Of course, given the strong effects on carbon allocation mediated by T6P there may need to be careful balancing of effects of variation in TPS and TPP genes in new lines to avoid trade‐offs. For example improvements in grain numbers could be offset by reduction in grain size. However, potentially better insight into such trade‐offs and the possibility of uncoupling them could be realized through targeting the genes associated with grain‐related traits. The link of T6P with sink‐led increase in photosynthesis (Oszvald et al., 2018) gives optimism that modifications of TPSs and TPPs can give rise not only to beneficial changes in partitioning within the plant but also to an overall increase in carbon assimilated due to enhanced sink capacity. Our work provides opportunities for the first time in a regulatory pathway in wheat. One way T6P may be having such a large effect on the reproductive tissue is through the regulation of *FT*‐genes (*FLOWERING LOCUS T*‐*like*). T6P regulates flowering time in Arabidopsis through *FT* (Wahl et al., 2013); in cereals, *FT* may have a broader role in productive development beyond flowering time including effects on spike development, spikelet number and fertility (Liu et al., 2019). This hypothesis can now be tested.

In conclusion, our study shows the importance of the trehalose pathway as a contributor to crop improvement both historically and for the future. The wealth of information will direct strategies of crossing and selection and in‐depth mode of action studies to better define the specific contribution of TPS and TPP genes to yield traits.

## CONFLICT OF INTEREST

The authors declare that the research was conducted in the absence of any commercial or financial relationships that could be construed as a potential conflict of interest.

## AUTHOR CONTRIBUTIONS

D.H.L performed statistical and quantitative genetic analysis. A.W, C.A.G and A.A.I contributed to the exome data analysis and interpretation of the biological results. G.M and M.R conducted the field experiment, assisted with the phenotypic data, and generated the 35 K SNP Chip data. R.J and A.H designed enrichment capture probe set and performed bioinformatics analysis. K.H.P assisted with the bioinformatics analysis. D.H.L and M.J.P. wrote the article, which all authors edited and approved.

## Supporting information

Supplementary Material

Table S1

## Data Availability

The exome capture data, as well as the R scripts used for most of the analyses in this study, can be found at https://github.com/DaniloLyra/exome_HiBAP_data. The genotypic (35 K SNP Chip) and phenotypic data are available in Molero et al., (2019).
